# Large-scale risk prediction applied to Genetic Analysis Workshop 17 mini-exome sequence data

**DOI:** 10.1186/1753-6561-5-S9-S46

**Published:** 2011-11-29

**Authors:** Gengxin Li, John Ferguson, Wei Zheng, Joon Sang Lee, Xianghua Zhang, Lun Li, Jia Kang, Xiting Yan, Hongyu Zhao

**Affiliations:** 1 Department of Epidemiology and Public Health, Yale University, 60 College Street, New Haven, CT 06520, USA; 2 Keck Laboratory, Yale University, 300 George Street, New Haven, CT 06511, USA; 3 Department of Electronic Science and Technology, University of Science and Technology of China, Hefei, China; 4 Bioinformatics and Molecular Imaging Key Laboratory, Huazhong University of Science and Technology, Wuhan, China

## Abstract

We consider the application of Efron’s empirical Bayes classification method to risk prediction in a genome-wide association study using the Genetic Analysis Workshop 17 (GAW17) data. A major advantage of using this method is that the effect size distribution for the set of possible features is empirically estimated and that all subsequent parameter estimation and risk prediction is guided by this distribution. Here, we generalize Efron’s method to allow for some of the peculiarities of the GAW17 data. In particular, we introduce two ways to extend Efron’s model: a weighted empirical Bayes model and a joint covariance model that allows the model to properly incorporate the annotation information of single-nucleotide polymorphisms (SNPs). In the course of our analysis, we examine several aspects of the possible simulation model, including the identity of the most important genes, the differing effects of synonymous and nonsynonymous SNPs, and the relative roles of covariates and genes in conferring disease risk. Finally, we compare the three methods to each other and to other classifiers (random forest and neural network).

## Background

The development of disease-risk prediction models based on genome-wide association data is a great challenge to statisticians. A major contributing factor to this difficulty is that the observed effects of the most significant features in any particular model are likely to be overestimates of their true effects [1]. Because of the complexities of a Bayesian analysis with hundreds of thousands of features, most of the shrinkage techniques that have been proposed to deal with this problem have a frequentist flavor, such as the LASSO (least absolute shrinkage and selection operator) and ridge regression [2]. Although these procedures tend to be computationally convenient, the resulting shrinkage could be considered ad hoc compared with an empirical Bayes alternative [3], because for the empirical Bayes alternative model shrinkage is guided directly by both the proportion of associated variants and the effect sizes for this subset of associated variants.

Genetic Analysis Workshop 17 (GAW17) provided a large-scale mini-exome sequence data set with a high proportion of rare variants. In this data set the number of genes far exceeds the number of samples, and, as a result, finding a good risk prediction model is a difficult challenge. Here, we demonstrate the use of an empirical Bayes algorithm, originally proposed by Efron [4] in a microarray case-control context, that is particular suitable to this large-scale data setup. This algorithm is a modified version of linear discriminant analysis (LDA) in which certain parameters, which represent standardized differences in the mean expression for case and control subjects, are shrunk before they are substituted into the LDA rule. In addition to describing some of the subtleties that need to be considered when applying Efron’s method to the GAW17 data (or other genome-wide association data), we develop two extensions that allow us to incorporate single-nucleotide polymorphism (SNP) annotation information into the prediction rule: the weighted empirical Bayes (WEB) model and the joint covariance (JC) model. To show the competitive performance of our proposed methods, we compare them with other classifiers: the random forest and the neural network.

## Methods

### Choice of gene score

A gene score is a composite value calculated by combining all SNP information within the same gene. Several advantages are gained by applying Efron’s empirical Bayes method to such gene scores instead of to individual SNPs. First, by pooling SNPs together in the correct way, we can potentially enrich the signal-to-noise ratio of the data. Second, the dimensionality of the feature space is greatly reduced (from 24,487 SNPs to 3,205 gene scores). Finally, even though LDA as a technique does not require the feature variables to be normal, it is actually an optimal procedure if they are. Although the number of rare alleles for a particular SNP cannot be considered a normal variable, applying this assumption to the score for genes that have many SNPs may be more reasonable.

Let *X_ij_* denote the Madsen-Browning gene score [5] that summarizes SNPs in gene *i* for individual *j*. This gene score is calculated as:(1)

where *G_lj_* is the number of rare variants for individual *j* at SNP *l*, *K* is the number of SNPs within gene *i*, and  is the empirical minor allele frequency (MAF) at SNP *l*. In practice, the Madsen-Browning method, which up-weights SNPs with a lower MAF when calculating gene scores, gives more coherent results on the GAW17 data, and whole gene scores are calculated based on this pooling method.

### Method 1: empirical Bayes method

We assume that there are *n*_1_ case subjects and *n*_2_ control subjects, where *n* is the total number of individuals; that is, *n* = *n*_1_ + *n*_2_. Suppose that there is no correlation between different gene scores; then the LDA rule is to classify an individual having *N* gene scores (*X*_1_, …, *X_N_*) as belonging to the disease or case group if:(2)

where(3)

and(4)

Here *μ_i_*_,1_ is the mean score for the *i*th gene in the case group, *μ_i_*_,2_ is the mean score for the *i*th gene in the control group, and *σ_i_* is the common standard deviation of the interindividual gene score values for gene *i* in either the case or control group. To apply such a method to real data, all the parameters in Eq. (2) must be estimated. If *σ_i_* is known, then the *Z* test statistic:(5)

where(6)

has expectation *δ_i_* and is approximately normally distributed. A naive application of LDA would assign an individual to the disease group if:(7)

where(8)

In practice, one would want to consider only genes with the largest *Z* statistics in application of Eq. (2), effectively restricting the range of the sum to the subset of the most associated genes. Unfortunately, a large selection bias is associated with using the *Z* statistics directly for this subset of genes, because they are most likely large overestimates of the true values of *δ_i_*. However, if we can assume that *Z_i_* is normally distributed with variance 1 (which is true asymptotically no matter what the distribution of the original *X_i_*), we can apply the empirical Bayes approach to obtain a Bayes estimate of *δ_i_* that will effectively shrink *Z_i_* toward zero using an empirically estimated prior distribution. These Bayes estimates of *δ_i_* can then be substituted for *Z_i_* in Eq. (7) to produce a better prediction rule, which assigns an individual to the disease group if:(9)

where *S* is the subset of genes showing the largest marginal association with the disease.

### Model 2: weighted empirical Bayes model

We expected that nonsynonymous SNPs are more likely to be directly involved in disease pathogenesis than synonymous SNPs. In this section, we propose a method to incorporate this annotation information into the empirical Bayes model. By fixing gene *i*, we separately consider two gene scores calculated by restricting the set of SNPs to contain only synonymous or only nonsynonymous SNPs. We denote these gene scores as  and , respectively. The relative importance of the nonsynonymous SNPs compared to the synonymous SNPs in gene *i* can be measured as:(10)

where *p_i_*_|_*_n_* and *p_i_*_|s_ are *p*-values associated with the *i*th gene score from the nonsynonymous SNPs and the synonymous SNPs, respectively. These *p*-values were calculated by fitting a logistic regression model in which the disease trait is regressed on either the synonymous or nonsynonymous gene and the Smoke covariate. A larger *w_i_* implies that the nonsynonymous SNPs from the *i*th gene have a relatively strong association with the disease trait compared with the synonymous SNPs. Throughout this section, the superscripts *n* and *s* refer to nonsynonymous and synonymous, respectively. The other notation is consistent with that introduced in the Model 1 subsection.

By combining the gene weight with the gene scores from both nonsynonymous SNPs and synonymous SNPs, we create a new gene score (weighted score):(11)

In this setting, the LDA rule is to classify an individual with new measurements () as belonging to the disease group if:(12)

where  and  are defined similarly as in the Model 1 subsection.

As before,  is estimated by shrinking  using the empirical Bayes method developed by Efron [4]. The test statistic:(13)

still follows a normal distribution with expectation  and variance 1; then the application of LDA would assign an individual in the same way.

### Model 3: joint covariance model

The strong linkage disequilibrium (LD) between nonsynonymous SNPs and synonymous SNPs for any particular gene may induce nonsynonymous SNPs and synonymous SNPs to be highly correlated. This correlation may greatly affect the eventual predicting result. Building a bivariate model to incorporate nonsynonymous and synonymous SNP information simultaneously will properly overcome this difficulty. More realistically, we can assume that:(14)

where *P_i_* is the correlation matrix for . Now define:(15)

and(16)

After some algebra, it follows that the optimal LDA rule is to assign an individual to the disease group if:(17)

Here we consider  and  to be different populations of parameters, and, as a result, the associated empirically estimated prior distributions should be different. This motivates shrinking the nonsynonymous and synonymous *Z* values separately and then applying the resulting Bayes estimates into Eq. (17). If there is evidence in the data that the nonsynonymous SNPs are more powerful in distinguishing between disease and nondisease, then the synonymous SNPs will be shrunk more. This implicitly gives the nonsynonymous gene scores higher weight in the prediction rule.

### Other issues: multiple replicates, treatment of covariates, and cross-validation and selection

One issue that the models need to take into account is multiple replicates. The GAW17 data are generated from a simulation model that assigns deleterious effects to some coding variants within a subset of genes in specific pathways from the 1000 Genomes Project [6]. A unique feature of the GAW17 data is that a large proportion of rare variants are reliably observed in most of the 200 replicates of the data set. Thus for any particular gene *i*, we can define *Z* statistics for *R* replicates {*Z_i_*_1_, …, *Z_iR_*}, each of which has an *N*(*δ_i_*, 1) distribution. One can then use:(18)

as a better estimate of *δ_i_*. However,  no longer has variance 1. A naive analysis would propose:(19)

However, one would expect that:(20)

because there should be a tendency for the sets of individuals having the disease phenotype for any two different replicates to have significant overlap. Under the assumption that:(21)

for the *s*th replicate and *t*th replicate for the *i*th gene,(22)

We can then standardize  appropriately as:(23)

Note that(24)

Because the new variables  have variance 1, Efron’s shrinkage algorithm can be applied directly to . Note that these shrunken *Z* values are the Bayes estimates of:(25)

where we define:(26)

The values:(27)

are then substituted into Eq. (9). To estimate *ρ*, we assume the relationship:(28)

which is true under the assumption Cor(*Z_is_*, *Z_it_*) = *ρ* for any *i*, *s*, and *t* and yields the estimate:(29)

The second issue in the models is the treatment of covariates. The covariates available in the GAW17 data (i.e., age, sex, and smoking status) have a dominant role in conferring disease risk, and it does not make sense to shrink these variables. When we allow covariates into our prediction rule, the prediction formula becomes:(30)

The last issue we want to mention is cross-validation and selection of the best subset of genes. Cross-validation is necessary to select the number of genes involved in any of the prediction rules to avoid the bias of prediction error. Cross-validation is implemented by using 50 replicates of the GAW17 data as training data, 50 replicates as test data, and the other 100 replicates as validation data. The *Z* scores and associated Bayes estimates are calculated on the training data. The error is evaluated on the test data using the prediction rule for each possible number of genes until we have clearly found the prediction rule with the minimum cross-validation error. The best prediction rule is finally applied to the validation data to find an unbiased estimate of the cross-validation error. The optimal number of genes to use in the prediction rule is calculated based on the prediction accuracy on the test data set. It should be noted that for the cross-validation we use a rule of the form:(31)

to account for the imbalance between case and control samples in the actual GAW17 data.

### Other classifiers

To evaluate the competitive performance of our proposed methods, we also fitted a random forest classifier [7] and a neural network classifier to the GAW17 data. The random forest classifier is known to perform remarkably well on a large variety of risk prediction problems (see [8]) and has been extensively used in genomic applications. The comparable performance to other classification methods, such as diagonal linear discriminant analysis (DLDA), *K* nearest neighbor (KNN) analysis, and support vector machines (SVM), has been demonstrated in a microarray study [9], and the successful application to a large data set has been demonstrated in a genome-wide association study [10]. The technique works by fitting a large number of classification or regression trees to bootstrapped versions of the original data set and then averaging over all these trees to form the prediction rule. The neural network classifier is another efficient learning method and has been widely used in many fields, especially risk prediction [8].

## Results

Table 1 displays the 10 most important variables that were found using the empirical Bayes (EB), weighted empirical Bayes (WEB), and joint covariance (JC) methods. It is clear that the environmental variables Age and Smoke have extremely strong signals and dominate the resultant models whenever they are included. In addition, the gene *FLT1* expresses a strong association with the disease trait and is found in the gene list for these three methods. We also detected another gene, *C10ORF107*, that is near to the true causal gene *SIRT1*. If we extend the gene list to the 30 most highly associated genes, *PIK3C2B*, another true causal gene, is involved in the prediction rule. Under the simulation design for the GAW17 data set, if a large proportion of rare variants are involved in this data set, then we need to record the number of SNPs and the minor allele frequency (MAF) interval of SNPs within these highly significant genes (see Table 1). It is obvious that the MAF of most SNPs within these selected genes is less than 0.01. Both the WEB and JC methods incorporate SNP annotation information in the models; the number of SNPs is further divided into two groups: the number of synonymous SNPs and the number of nonsynonymous SNPs. When compared with the synonymous SNPs, the important genes in Table 1 have a larger proportion of nonsynonymous rare variants in the WEB and JC models.

**Table 1:** Prediction rule of three proposed methods

Feature	Empirical Bayes method			Weighted empirical Bayes method				Joint covariance model			
	
	Genes	#SNP	MAF	Genes	#Syn SNP	#Non SNP	MAF	Genes	#Syn SNP	#Non SNP	MAF
1	**Age**			**Age**				**Age**			
2	**Smoke**			**Smoke**				**Smoke**			
3	*ATP11A*	1	0.29	*SUSD2*	13	23	<0.01	*ATP11A*	1		0.29
					2	4	0.01–0.05				
					1	2	≥0.05				
4	***FLT1***	25	<0.01	***FLT1***	8	17	<0.01	*BUD13*	1		0.11
		7	0.01–0.05		5	2	0.01–0.05				
		3	≥0.05		2	1	≥0.05				
5	*SUSD2*	36		*ATP11A*	1		0.29	***C10ORF107***	1		0.13
		6									
		3									
6	*BUD13*	1	0.11	*RIPK3*	4	13	<0.01	*RIPK3*	4	13	<0.01
					1	1	0.01–0.05		1	1	0.01–0.05
					1	1	≥0.05		1	1	≥0.05
7	*RIPK3*	17	<0.01	*BUD13*	1		0.11	*SUSD2*	13	23	<0.01
		2	0.01–0.05						2	4	0.01–0.05
		2	≥0.05						1	2	≥0.05
8	***C10ORF107***	1	0.13	*ADAMTS4*	10	23	<0.01	***FLT1***	8	17	<0.01
					2	2	0.01–0.05		5	2	0.01–0.05
					1	2	≥0.05		2	1	≥0.05
9	*ADAMTS4*	33	<0.01	*WNT16*	8	7	< 0.01	*GPR158*		1	0.1
		4	0.01–0.05		1	2	0.01–0.05				
		3	≥0.05			2	≥0.05				
10	*MAP3K12*	14	<0.01	*GOLGA1*	1		<0.01	*ANAPC5*	14	12	<0.01
		3	0.01–0.05			1	0.01–0.05		1		0.01–0.05
						1	≥0.05				≥0.05

Top 10 important features from the model incorporating genes and environmental variables for the three proposed methods. #SNP, number of SNPs within a specific gene; #Syn SNP, number of synonymous SNPs; #Non SNP, number of nonsynonymous SNPs. MAF shows three intervals of minor allele frequency: MAF < 0.01, 0.01 ≤ MAF < 0.05, and MAF ≥ 0.05. The boldfaced genes and environmental variables are real causal features that are selected across the three proposed models.

The feature selection procedure of the EB method is also compared with the random forest (RF) method and logistic regression (LR). The comparison results are summarized in Table 2. According to the RF classifier, 10 features with the largest sum importance score are selected from separate RF classifiers on each of the 100 replicates. Under LR, 10 features with the smallest *p*-values are chosen from the 100 replicates. In brief, six features in the RF method and 10 features in LR are consistent with features in the EB model, and the concordance rate in feature selection is quite high between our proposed methods and other classifiers.

**Table 2:** Comparison of the prediction rule between the empirical Bayes and other classifiers

Feature	Empirical Bayes method			Random forest classifier			Logistic regression		
	
	Genes	#SNP	MAF	Genes	#SNP	MAF	Genes	#SNP	MAF
1	**Age**			**Age**			**Age**		
2	**Smoke**			**Smoke**			**Smoke**		
3	*ATP11A*	1	0.29	***FLT1***	25	<0.01	*SUSD2*	36	<0.01
					7	0.01–0.05		6	0.01–0.05
					3	≥0.05		3	≥0.05
4	***FLT1***	25	<0.01	*SUSD2*	36	<0.01	*ATP11A*	1	0.29
		7	0.01–0.05		6	0.01–0.05			
		3	≥0.05		3	≥0.05			
5	*SUSD2*	36		*SHD*	10	< 0.01	*BUD13*	1	0.11
		6			1	0.01–0.05			
		3			2	≥0.05			
6	*BUD13*	1	0.11	*RIPK3*	17	<0.01	*RIPK3*	17	<0.01
					2	0.01–0.05		2	0.01–0.05
					2	≥0.05		2	≥0.05
7	*RIPK3*	17	<0.01	*ADAMTS4*	23	<0.01	***FLT1***	25	<0.01
		2	0.01–0.05		4	0.01–0.05		7	0.01–0.05
		2	≥0.05		3	≥0.05		3	≥0.05
8	***C10ORF107***	1	0.13	*CECR1*	8	<0.01	*MAP3K12*	14	<0.01
						0.01–0.05		3	0.01–0.05
					4	≥0.05			≥0.05
9	*ADAMTS4*	33	<0.01	*GOLGA1*	1	<0.01	*ADAMTS4*	33	<0.01
		4	0.01–0.05		1	0.01–0.05		4	0.01–0.05
		3	≥0.05		1	≥0.05		3	≥0.05
10	*MAP3K12*	14	<0.01	*C14orf108*	16	<0.01	***C10ORF107***	1	0.13
		3	0.01–0.05		1	0.01–0.05			
					2	≥0.05			

The top 10 important features from the model incorporating genes and environmental variables between our proposed method (empirical Bayes) and other classifiers (random forest and logistic regression). #SNP, number of SNPs within a specific gene. MAF shows three intervals of minor allele frequency: MAF < 0.01, 0.01 ≤ MAF < 0.05, and MAF ≥ 0.05. The boldfaced genes are real causal features that are selected simultaneously from the three models; for example, *FLT1* is observed using the three classifiers.

The comparison results of misclassification error for our proposed methods are displayed in Table 3. The first row in Table 3 gives the average misclassification error obtained from the model derived on the training and test data to predict the phenotype values of the 100 validation replicates (see the earlier discussion of cross-validation). Note that this error may depend on which 100 replicates are chosen. To explore this issue, we randomly split the 200 replicates into training, test, and validation sets five times. This enabled us to compute a standard error of the mean prediction error for the EB, WEB, and JC methods (see Table 3). Note that the differences between the means are large relative to the standard errors and likely reflect true differences in the performance of the three methods. It is clear that the WEB method provides the smallest average misclassification error (0.236) followed by the JC method (0.241) and the EB method (0.26).

**Table 3:** Cross-validation error and AUC value for the three methods

Item	Model	Statistic	Empirical Bayes method	Weighted empirical Bayes method	Joint covariance model
Cross-validation error	Gene + environment	Mean	0.26	0.24	0.24
		SE	0.0020	0.0011	0.0012
AUC value	Gene + environment	Mean	0.76	0.80	0.78
		SE	0.0102	0.0015	0.0148
AUC value	Gene	Mean	0.60	0.64	0.62
		SE	0.0191	0.0183	0.0191

AUC is the area under the ROC curve when minimizing the cross-validation error. SE, standard error of the cross-validation error and the AUC value.

**Figure 1:**
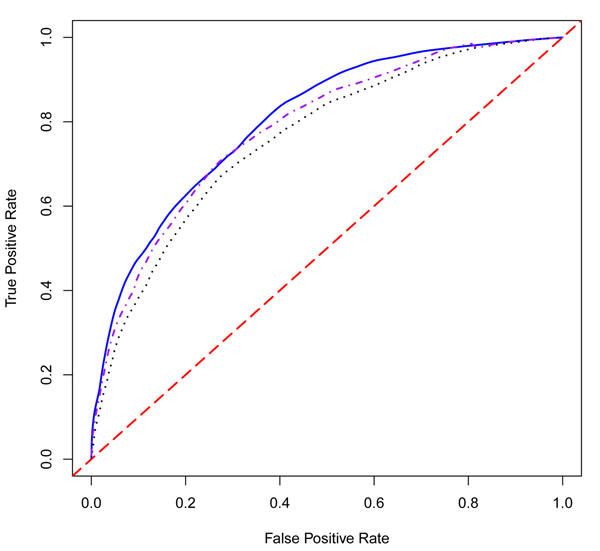
**ROC curves for the EB, WEB, and JC methods for the prediction model using genes and environmental covariates.** The black dotted line is the ROC curve generated from gene and environmental covariates in the prediction model based on the empirical Bayes (EB) method. The blue solid line is the ROC curve from the weighted empirical Bayes (WEB) model. The purple dot-dashed line is the ROC curve from the joint covariance (JM) model. The red dashed line is the diagonal.

We also compared the prediction accuracies for our proposed methods using the area under curve (AUC) value (Table 3). When both genes and environmental variables are involved in the prediction model, the WEB method gives the highest AUC value (0.80) followed by the JC method (0.78) and the EB method (0.76). All three methods perform better than other classifiers: RF (0.67), neural network 1 (NN1: 0.68), and neural network 2 (NN2: 0.70) (Table 4). It is easy to see that the EB-based neural network classifier (0.70) provides a larger AUC value than the LR-based neural network classifier (0.68). The relevant three receiver operating characteristic (ROC) curves corresponding to our proposed methods are plotted in Figure 1.

**Table 4:** Comparison of AUC value for the empirical Bayes and other classifiers

Item	Model	Statistics	Empirical Bayes model	Random forest classifier	Neural network 1	Neural network 2
AUC value	Gene + environment	Mean	0.76	0.67	0.68	0.70
		SE	0.0102	–	–	–

AUC value indicates the area under the ROC curve when minimizing the cross-validation error. Neural network 1 used selected features from the logistic regression; neural network 2 used selected features from the empirical Bayes method. SE is the standard error of the AUC value.

**Figure 2:**
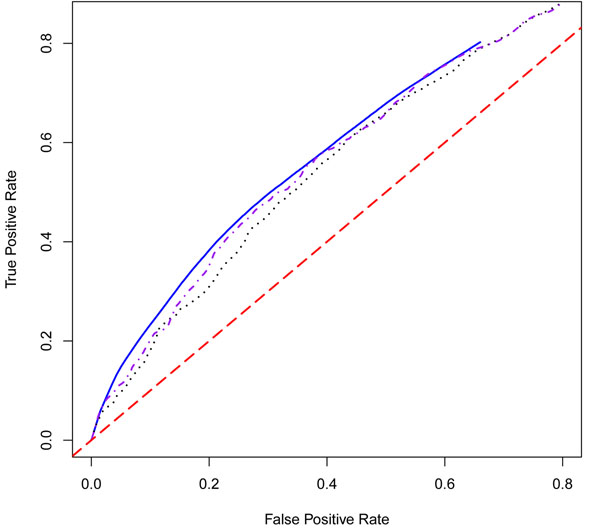
**ROC curves for the EB, WEB and JC methods for the prediction model using genes only**. The black dotted line is the ROC curve generated from the prediction model using genes only, based on the empirical Bayes (EB) method. The blue solid line is the ROC curve from the weighted empirical Bayes (WEB) model. The purple dot-dashed line is the ROC curve from the joint covariance (JC) model. The red dashed line is the diagonal.

In summary, our proposed methods significantly improve the accuracy of the prediction model compared with other classifiers. Because the environmental variables have such a strong influence in the prediction model, we also fitted the EB, WEB, and JC models using the genetic variables alone in order to determine the prediction accuracy achievable through purely genetic information (Table 3). In this case, the best AUC value is achieved using the WEB method (0.64) followed by the JC method (0.62) and the EB method (0.60) (Figure 2).

Of course, in practical applications more than one replicate cannot be obtained. This scenario can be represented by training and testing the prediction model using only one replicate. When one does this, the prediction model based on the EB method is still quite good. For example, *FLT1* is always in the list of the 10 most strongly associated features in the EB model. If a similar model is fitted using the RF classifier, no causal genes tend to be found in the top gene list (Table 5). In addition, the EB method provides a substantively larger AUC value (0.72) than the RF classifier (0.66) (Table 6).

**Table 5:** Prediction rule for two classifiers based on one replicate

Feature	Empirical Bayes classifier			Random forest classifier		
	
	Genes	#SNP	MAF	Genes	#SNP	MAF
1	**Age**			**Age**		
2	**Smoke**			**Smoke**		
3	*GOLGA1*	1	<0.01	*OR1L6*		<0.01
		1	0.01–0.05		3	0.01–0.05
		1	≥0.05		1	≥0.05
4	***FLT1***	25	<0.01	*VTI1B*	9	<0.01
		7	0.01–0.05		1	0.01–0.05
		3	≥0.05		1	≥0.05
5	*NFKBIA*	6	<0.01	*DENND1A*	19	<0.01
			0.01–0.05		3	0.01–0.05
		2	≥0.05		4	≥0.05
6	*DGKZ*	17	<0.01	*C9ORF66*	4	<0.01
		4	0.01–0.05		3	0.01–0.05
		1	≥0.05		4	≥0.05
7	*SMTN*	23	<0.01	*CECR1*	8	<0.01
		4	0.01–0.05			0.01–0.05
		2	≥0.05		4	≥0.05
8	*PAK7*	1	0.30	*MAP3K12*	14	<0.01
					3	0.01–0.05
						≥0.05
9	*ADAM15*	22	<0.01	*SLC20A2*	24	<0.01
		5	0.01–0.05		4	0.01–0.05
		3	≥0.05		1	≥0.05
10	*ADAMTS4*	33	<0.01	*ALK*	9	<0.01
		4	0.01–0.05		1	0.01–0.05
		3	≥0.05		6	≥0.05

Top 10 important features from the model incorporating genes and environmental variables (Age and Smoke) using one replicate between our proposed method (empirical Bayes) and the random forest method. #SNP, number of SNPs within a specific gene. MAF shows three intervals of minor allele frequency: MAF < 0.01, 0.01 ≤ MAF < 0.05, and MAF > 0.05. The boldfaced gene FLT1 still can be selected in the empirical Bayes method but is not observed using the random forest method.

**Table 6:** Cross-validation error and AUC value for the empirical Bayes and random forest methods based on one replicate

Item	Model	Statistics	Empirical Bayes method	Random forest method
Cross-validation error	Gene + environment	Mean	0.26	0.23
		SE	0.009	–
AUC value	Gene + environment	Mean	0.72	0.66
		SE	0.058	–

AUC value is the area under the ROC curve when minimizing the cross-validation error. SE is the standard error of the cross-validation error and the AUC value.

## Conclusions

It is well known that developing a good disease risk prediction model based on genome-wide association data is a difficult task; the number of predictors can be orders of magnitude higher than the number of samples that are genotyped. This is certainly the case in the GAW17 mini-exome data set, in which there is information on 24,487 SNPs for only 697 samples. In this paper, we have used the good properties of the empirical Bayes prediction model that Efron [4] developed in a large-scale microarray context to build a prediction model for these data. An interesting feature of the GAW17 data is that they provide annotation information for each SNP in the form of a synonymous/nonsynonymous indicator. Because only nonsynonymous SNPs affect protein function, we expect that they, rather than synonymous SNPs, are more likely to be directly involved in disease pathogenesis. We propose two ways (weighted empirical Bayes model and joint covariance model) to properly incorporate this annotation information into the prediction model. The weighted empirical Bayes model provides the best performance (relatively small cross-validation error and larger AUC value). We also compare the three EB classifiers with two other popular classifiers (random forest and neural network). The EB classifiers have superior prediction performance in terms of AUC value. Based on this analysis, we think that Efron’s empirical Bayes risk prediction model, extended in the manner that we describe here, is a useful and powerful tool for disease risk prediction in genome-wide association studies.

## Competing interests

The authors declare that there are no competing interests.

## Authors’ contributions

GL carried out the design of models, data analysis, and wrote the draft, JF carried out the design of models and wrote the manuscript, WZ participated in preparing the gene score data and performed random forest analysis, JSL, XZ and LL participated in preparing the gene score data, JK participated in the comparison results between our proposed methods and other classifiers, XY participated in the progression of studies, HZ managed the progression of this project and reviewed the draft. The final manuscript has been approved by all authors after they read it.

## References

[B1] Zhong H Prentice RL Bias-reduced estimators and confidence intervals for odds ratios in genome-wide association studies Biostatistics 2008 9 621 634 10.1093/biostatistics/kxn001 18310059 PMC2536726

[B2] Tibshirani R Regression shrinkage and selection via the Lasso J R Stat Soc B 1996 58 267 288

[B3] Robert C The Bayesian Choice 2001 2nd New York, Springer Texts in Statistics

[B4] Efron B Empirical Bayes estimates for large-scale prediction problems J Am Stat Assoc 2009 104 1015 1028 10.1198/jasa.2009.tm08523 20333278 PMC2844005

[B5] Madsen BE Browning SR A groupwise association test for rare mutations using a weighted sum statistic PLoS Genet 2009 5 e1000384 doi:10.1371/journal.pgen.1000384 10.1371/journal.pgen.1000384 19214210 PMC2633048

[B6] Almasy L Dyer TD Peralta JM Kent JW Jr Charlesworth JC Curran JE Blangero J Genetic Analysis Workshop 17 mini-exome simulation BMC Proc 2011 5 suppl 8 S2 22373155 10.1186/1753-6561-5-S9-S2 PMC3287854

[B7] Breiman L Random forests Machine Learning 2001 45 5 32 10.1023/A:1010933404324

[B8] Hastie T Tibshirani R Friedman J The Elements of Statistical Learning: Data Mining, Inference, and Prediction 2009 2nd New York, Springer Series in Statistics

[B9] Diaz-Uriarte R Alvarez de Andres Gene selection and classification of microarray data using random forest BMC Bioinformatics 2006 7 3 10.1186/1471-2105-7-3 16398926 PMC1363357

[B10] Goldstein BA Hubbard AE Cutler A Barcellos LF An application of random forests to a genome-wide association data set: methodological considerations and new findings BMC Genet 2010 11 49 20546594 10.1186/1471-2156-11-49 PMC2896336

